# Reply to Jarvis and Chong: Understanding punishment insensitivity phenotypes using computational modelling

**DOI:** 10.1073/pnas.2316107120

**Published:** 2023-10-31

**Authors:** Philip Jean-Richard-dit-Bressel, Gavan P. McNally

**Affiliations:** ^a^School of Psychology, University of New South Wales, Sydney, NSW 2052, Australia

When exposed to a straightforward task where an action has a net detrimental outcome, we have found that the propensity to stop making detrimental choices is bimodally distributed across humans (1, 2) and other animals (3). A question is what psychological processes drive so many to persist in behaviors that are unfavorable. We found that robust, highly clustered differences in avoidance behavior (“Sensitive”, “Unaware”, “Compulsive”) were attributable to differences in what participants learned about their actions: Failures to avoid were accompanied by pronounced failures in recognising the connection between specific actions and their consequences. While this has been used to highlight an overlooked cognitive cause for the persistence of suboptimal behavior (4), these studies provide a qualitative inference and do not specify computations that drive disparate behavior.

Using a modified reinforcement learning model (Opponent actor learning [OpAL] (5, 6)), Jarvis and Chong (7) show that phenotypic behavior and effects of different punishment probabilities can be emulated through gamma-distributed variations in a single computational parameter: an exponential no-go weighting parameter that determines how acquired knowledge is translated into behavior. They also recapitulate the resistance of Compulsive individuals to beneficial information if this information was simulated as a 10-fold increase in the inverse temperature parameter (shifting participants toward an exploit policy on the explore–exploit continuum).

This computational architecture raises important questions. For example, if the key difference between phenotypes is a computational parameter acting upon how existing knowledge is used rather than on how that knowledge is acquired (i.e., is a gatekeeper of beliefs→behavior, not experience→beliefs), it is unclear why Unawares and Compulsives could not accurately report Action–Punisher relationships and why reported Action–Punisher beliefs corresponded so strongly to behavior (at least for Sensitives and Unawares). This issue also pertains to how contingency information is modeled as a change in the temperature parameter, another gatekeeper between beliefs and behavior, without changes to contingency knowledge (Action–Punisher beliefs).

This conceptual disjunct between computational and cognitive models is a healthy and welcome result of different approaches interacting with the same empirical findings. Perhaps this disjunct is a category error, with the veil between subconscious computations and conscious beliefs existing further along the computational pipeline (i.e., after no-go weights are applied in the model). This raises questions about how static this weighting parameter might be, as it has far-reaching implications for the tractability of beliefs and behavior. At the other extreme, this disjunct could be an example of a model possessing excellent predictive validity while having construct validity issues (8), potentially stemming from extant difficulties with diagnosing learning mechanisms from the shapes of learning curves alone (9). These possibilities require further examination, and the iterative interplay between hypothesis-driven experimentation, computational approaches, and theory development will undoubtedly be a fruitful way forward to understanding adaptive versus maladaptive responses to detriment (8).

## References

[1] P. Jean-Richard-dit-Bressel , A cognitive pathway to punishment insensitivity. Proc. Natl. Acad. Sci. U.S.A. **120**, e2221634120 (2023). 37011189 10.1073/pnas.2221634120 PMC10104546

[2] P. Jean-Richard-dit-Bressel , Punishment insensitivity in humans is due to failures in instrumental contingency learning. Elife **10**, e69594 (2021). 34085930 10.7554/eLife.69594 PMC8177883

[3] P. Jean-Richard-Dit-Bressel, C. Ma, L. A. Bradfield, S. Killcross, G. P. McNally, Punishment insensitivity emerges from impaired contingency detection, not aversion insensitivity or reward dominance. Elife **8**, e52765 (2019). 31769756 10.7554/eLife.52765 PMC6890457

[4] G. P. McNally, P. Jean-Richard-dit-Bressel, E. Z. Millan, A. J. Lawrence, Pathways to the persistence of drug use despite its adverse consequences. Mol. Psychiatry 1–10 (2023). 10.1038/s41380-023-02040-z PMC10611585 36997610

[5] A. G. Collins, M. J. Frank, Opponent actor learning (OpAL): Modeling interactive effects of striatal dopamine on reinforcement learning and choice incentive. Psychol. Rev. **121**, 337 (2014). 25090423 10.1037/a0037015

[6] A. Jaskir, M. J. Frank, On the normative advantages of dopamine and striatal opponency for learning and choice. Elife **12**, e85107 (2023). 36946371 10.7554/eLife.85107 PMC10198727

[7] H. C. Jarvis, T. T.-J. Chong, A computational architecture of punishment insensitivity. Proc. Natl. Acad. Sci. U.S.A. **120**, e2312950120 (2023).3790664210.1073/pnas.2312950120

[8] I. Grahek, M. Schaller, J. L. Tackett, Anatomy of a psychological theory: Integrating construct-validation and computational-modeling methods to advance theorizing. Perspect. Psychol. Sci. **16**, 803–815 (2021). 33404380 10.1177/1745691620966794

[9] C. R. Gallistel, S. Fairhurst, P. Balsam, The learning curve: Implications of a quantitative analysis. Proc. Natl. Acad. Sci. U.S.A. **101**, 13124–13131 (2004). 15331782 10.1073/pnas.0404965101 PMC516535

